# Marginal kidney donor

**DOI:** 10.4103/0970-1591.33726

**Published:** 2007

**Authors:** Ganesh Gopalakrishnan, Siva Prasad Gourabathini

**Affiliations:** Department of Urology, Christian Medical College, Vellore, India

**Keywords:** Complex living donor, deceased marginal donor, marginal kidney donor, non-heart-beating donor

## Abstract

Renal transplantation is the treatment of choice for a medically eligible patient with end stage renal disease. The number of renal transplants has increased rapidly over the last two decades. However, the demand for organs has increased even more. This disparity between the availability of organs and waitlisted patients for transplants has forced many transplant centers across the world to use marginal kidneys and donors. We performed a Medline search to establish the current status of marginal kidney donors in the world. Transplant programs using marginal deceased renal grafts is well established. The focus is now on efforts to improve their results. Utilization of non-heart-beating donors is still in a plateau phase and comprises a minor percentage of deceased donations. The main concern is primary non-function of the renal graft apart from legal and ethical issues. Transplants with living donors outnumbered cadaveric transplants at many centers in the last decade. There has been an increased use of marginal living kidney donors with some acceptable medical risks. Our primary concern is the safety of the living donor. There is not enough scientific data available to quantify the risks involved for such donation. The definition of marginal living donor is still not clear and there are no uniform recommendations. The decision must be tailored to each donor who in turn should be actively involved at all levels of the decision-making process. In the current circumstances, our responsibility is very crucial in making decisions for either accepting or rejecting a marginal living donor.

The end stage renal disease (ESRD) population is increasing worldwide and has doubled in the Western world during the past decade.[1] End stage renal disease patients either remain on dialysis or undergo renal transplantation. Renal transplantation is better than dialysis for various reasons. In India about 80,000 patients are added annually to the pool of ESRD, however, only 2.4% undergo transplant.[2] This low transplantation rate in India is due to the lack of an appropriate cadaver transplant program and the inability of many to afford a transplant due to their socioeconomic status. In recent years the numbers of live donation surpassed cadaveric donations worldwide.[3] According to the UNOS scientific renal transplant registry, the number of patients on waiting lists for kidney transplantation increase by 10% annually whereas, the annual increase in the number of renal transplants is only 4%.[4] There is a significant demand for renal allografts. The average waiting period for cadaveric graft is more than five years in the US.[5] Annually 7% of the waiting list candidates die and the mortality rates decrease dramatically after transplantation. Efforts are made to increase the availability of organs for donation and usage of ‘Expanded-criteria donors’ is one of them.

The terms- ‘Expanded-criteria donors’, ‘Marginal kidney donor’ are not yet clearly defined. It simply means usage of suboptimal quality cadaveric renal grafts, non-heart-beating donors (NHBD) and living donors with some acceptable medical risks. In the United States, the discarded kidney rates were 7% and 12% in 1994 and 2003 respectively.[6] The most common reasons quoted for discard were poor quality, small size of the kidney and older donors.[7] With the increase in demand for allografts, many centers worldwide used marginal kidneys with informed consent. Although the short- and long-term outcomes with such grafts were inferior to normal criteria kidneys, it had significant survival advantage over the waitlisted dialysis patients.[8] Poor long-term outcome may be the consequence of an imbalance between the number of viable nephrons supplied and the metabolic demand of the recipient. The gap becomes wider when marginal kidneys are transplanted after prolonged cold ischemia.

## Types of marginal donor

1. Complex living donor
2. Non-Heart-beating donor (NHBD)
3. Deceased or cadaveric donor

## COMPLEX LIVING KIDNEY DONOR

The International Forum on the care of the Live Kidney donor was held in April 2004 at Amsterdam.[9] Over 100 transplant experts from more than 40 countries participated. The objective was to develop international consensus on the standard of care and define the responsibility of the transplant community for the live kidney donor. They emphasized that 50 years after the first successful live donor renal transplant there is enough published evidence showing only little long-term medical risk to a healthy donor after nephrectomy. However, the potential under-reporting of donor complications by the transplant team for various reasons should be kept in mind while interpreting the published literature. The term ‘Marginal living donor’ was used by Matas.[10] However, the term can be confusing, as the marginal deceased donor emphasizes on the graft being marginal and the only affects recipient outcomes. In the marginal living donor, focus is on the potential harm to the donor. Steiner[11] used the term ‘IMA donors’ for donors with isolated medical abnormalities. But multiple risk factors for future kidney disease may coincide in the same patient. The term ‘Complex living donor’ used by Resse[12] is probably preferred for all suboptimal donors where decision-making is a problem due to lack of sound medical data or consensus guidelines. He further categorized complex living donors based on certain risk factors [Table 1].

**Table 1:** Risk factors associated with complex living donor

Type of risk factor	Example
• Evidence of current renal disease	Hematuria, proteinuria, nephrolithiasis
• Direct risk for CKD	Hypertension, obesity
• Reduced nephron mass	Age ≥65 years
• Genetic risk factor	Family h/o of ESRD in 1st relative
• Risk factor for a CKD	Diabetes in a first-degree relative, Impaired fasting glucose
• Cardiovascular risk factor	Smoking, hyperlipidemia, hypertension
• Other	Black race, sickle trait
• Combination of previous risk factors	Hypertensive black patient

There is enough evidence to suggest that ideal living donors do not face risks for ESRD any higher than those of age-matched peers,[13,14] but this evidence cannot be applied to the complex donors. In fact, the emphasis should be to answer the risk of complex living donor developing CKD itself rather than ESRD.

### Elderly living donors

The definition of “normal” GFR changes with age and it decreases over time.[15–17] The decrease in GFR is approximately 1 ml/min/1.73 m^2^ per year after age 40. There is an acute decrease in GFR of approximately 30% after unilateral nephrectomy; however, the impact of unilateral nephrectomy on this rate of decline in GFR is unknown. Acceptable GFR in a donor is that which can be predicted to provide adequate function for both donor and recipient after donor nephrectomy/transplantation. A GFR of ≥80ml/min is generally considered as accepted value although some centers use ≥60ml/min. GFR corrected to the age rather than age itself determines acceptability for donation.

The poorer outcome of older donor age on deceased donor renal transplant was used to predict results in potential older living donors. The influence of donor age on the outcome of living donor kidney transplantation is not clear. Kumar *et al*.[18] retrospectively compared the long-term outcomes of 112 recipients of kidneys from elderly (>55 years) living related donors with 87 recipients who had younger donors (<45 years). No differences in graft and patient survival between the two groups were detected at one year and five years after transplantation. There was no additional morbidity or deterioration of preoperative blood pressure and renal function at one year in the elderly donors. Similar results were reported from the Mayo Clinic; the outcomes of 52 recipients of older (>50 years) living donor grafts were compared with a matched group of 103 recipients of younger (<50 years) donor kidneys.[19] Overall graft survival, patient survival and death-censored graft survival at three years did not differ significantly between the two groups. However, contrary to the above findings, significantly poorer survival of grafts from five years post-transplantation onwards was noted in a Japanese study of 343 older (>60 years) living donor allografts.[20] Similarly, Prommool *et al*. found donor age to be the most important risk factor for graft loss after the first five years.[21] In an analysis of their entire living donor population of 2,540 kidney transplants at the University of Minnesota, Matas and colleagues identified donor age greater than 55 years to be a significant risk factor for late graft loss.[22,23] The inferior outcomes of an older kidney graft might be a function of the anatomical and physiological changes that occur during aging.[24] Rates of short-term morbidity and mortality do not seem to be higher for elderly donors, but no data on long-term outcomes for this specific group are available.

### Hypertensive living donors

Blood pressure (BP) should preferably be measured by ambulatory blood pressure monitoring (ABPM). Patients with a BP ≥140/90 mmHg by ABPM are generally not acceptable as donors. Some patients with easily controlled hypertension who meet other defined criteria (e.g., ≥50 years of age, GFR ≥80 ml/min and urinary albumin excretion <30 mg/day) may represent a low-risk group for development of kidney disease after donation and may be acceptable as kidney donors. Steiner[25] calculated that the lifetime risk of ESRD in isolated mild to moderate hypertensives in the US as less than one in 100. However, this estimate is on baseline two-kidney risk for ESRD, irrespective of donation.

There is an increase in age-specific prevalence of hypertension in male donors unlike female donors.[9] Although they have been studied extensively, many centers do not accept hypertensive donors. The study by Textor *et al*.[26] reported 12 months of follow-up of GFR, proteinuria and blood pressure in 24 hypertensive and 125 non-hypertensive donors at the Mayo clinic. At donor nephrectomy and one year later, the hypertensive donors had a slightly lower GFR and slightly more proteinuria than non-hypertensive donors. However, the anti-hypertensives used and their effects on proteinuria was not controlled in the study. Our policy is to accept a hypertensive donor, if age was over 50 years, blood pressure controlled on a single antihypertensive agent and there was no target organ damage.

### Living donors with diabetes

Diabetes is associated with an increased risk of post-surgical complications and future development of renal failure compared to the general population. Evidence for increased risk of developing diabetic nephropathy after nephrectomy is available in experimental studies only.[27] Silveiro *et al*.[28] suggested that nephrectomy in a patient with Type 2 diabetes might increase the progression of disease and microalbuminuria. High risk for developing Type 2 diabetes includes those with a familial history, a BMI of ≥30 kg/m2, women with gestational diabetes and excessive alcohol use. Relatives of patients with diabetic nephropathy (Type I) appear to be at increased risk of developing diabetic nephropathy. Seaquist *et al*.[29] presented data on diabetic siblings of insulin-dependent diabetics with and without end stage renal failure. Only 17% of siblings of subjects without nephropathy developed albuminuria, but most of the siblings of patients with diabetic nephropathy developed either albuminuria (41%) or end stage renal failure (41%). This data is really worrying and one cannot accept any sibling of insulin-dependent diabetes mellitus as a donor. All other potential living kidney donors related to recipients with diabetes may have a preexisting increased risk of developing diabetes and diabetic nephropathy,[30] but the risk is not quantified. Simmons *et al*.[30] suggested that there are four major predictors of future diabetes—ethnic group, previous gestational diabetes, a high titer of islet cell antibody (for insulin-dependent diabetes mellitus) and impaired glucose tolerance. This again cannot be generalized for all ethnic groups all over the world. As Level I and II evidence is not available, only guidelines were issued at the International forum for living donor care. Individuals with a history of diabetes or fasting blood glucose of ≥126mg/dl (7.0mmol/L) on at least two occasions (or 2-h glucose with OGTT ≥ 200mg/dl (11.1mmol/L)) should not donate.[9] No comments were made regarding the acceptance of any related donor of a diabetic nephropathy individual.

### Living donors with nephrolithiasis

Nephrolithiasis may be discovered incidentally or with past history of stone disease. Two published case series addressed nephrolithiasis recurrence rates in patients after non-donor nephrectomy. In a series of 50 patients who underwent nephrectomy for complications related to a previous stone, Lee *et al*.[31] reported a recurrence rate for kidney stones of 30% in the contralateral kidney over a mean follow-up time of >five years. Two patients experienced anuric renal failure from recurrent stones and another patient developed ESRD. Worcester *et al*.[32] compared stone recurrence in 115 patients who underwent nephrectomy for various reasons (30% related to stones, 25% because of infection, 30% because of obstruction) *versus* 3151 patients with two kidneys. Recurrence was 14% in the patients who underwent nephrectomy, with a mean follow-up of six to eight years. He observed that stone recurrence in two-kidney patients was higher than in single-kidney patients but did not report the analogous recurrence rate for two-kidney patients during the same follow-up period. These patient populations likely have a higher risk for recurrent stones than would kidney donors with nephrolithiasis. Nonetheless, the lack of data on living donors with nephrolithiasis complicates extrapolation of these data to long-term donor risks. It therefore is understandable as to why there has been a reluctance to accept living donors with nephrolithiasis for kidney donation. Nevertheless kidneys with renal stones were knowingly transplanted.[33,34] There is hence a rethink on this problem.

An asymptomatic potential donor with no history of calculuria or colic even is found to have a single stone on evaluation may be suitable for kidney donation if:[9]

- No metabolic abnormality or urinary infection exists and if multiple stones or nephrocalcinosis are not evident on computed tomography (CT)
  Asymptomatic potential donor with current single stone may be suitable if: [9]
- The donor meets the criteria shown previously for single stone formers and current stone is ≤1.5 cm in size or potentially removable during transplant.

The data of Antony *et al*.[35] of five cases at three years’ follow-up is uneventful. The Leadbetter-Politano technique of ureteric reimplantation was recommended at surgery. It helps in easy ureteroscopic management of stone disease if required in future. The younger the donor age (age 25-35), the longer the exposure to the possibility of a recurrence.[32] It is not known whether stone formers who donate a kidney develop worse outcomes than those with two kidneys. However, a recurrent stone may not affect the function of a remaining kidney if it is carefully monitored.[9] One should keep in mind that patients with stone disease as such have lower renal function and a long-term vigilant follow-up for both donor and recipient is essential to accurately determine the outcome. We also believe that the above criteria are to be strictly followed in using stone-bearing kidneys from living donors for transplantation.

### Living donors with obesity

Obesity was defined by a body mass index (BMI) of ≥30 kg/m^2^. Increased body mass index (BMI) has been associated with the risk for proteinuria and FSGS and kidney function for obese donors can also be harmed indirectly through increased rates of diabetes, hypertension and the metabolic syndrome.[36] Heimbach *et al*.[37] compared obese and nonobese living donors at the Mayo clinic. Of 553 donors reviewed, 58% had a one-year follow-up data, at which point obese donors had similar levels of urinary microalbumin and GFR as other donors and no change in BMI. A cross-sectional study by Praga *et al*.[38] reported rates of proteinuria and CKD among patients who underwent non-donor nephrectomy at a single center in Spain. Nephrectomy had been performed for reasons such as nephrolithiasis, unilateral hydronephrosis or masses and complications of pyelonephritis. Exclusion criteria for the study were subsequent development of systemic diseases such as diabetes since nephrectomy. Of 115 patients initially considered, 73 were included, 20 of whom developed proteinuria. The proteinuric patients were more likely to be obese than nonproteinuric patients. A BMI of <35 Kg/m^2^ without comorbidity is acceptable for donation and encouraged to lose weight prior to kidney donation.[9] With laparoscopic donor nephrectomy becoming the standard of care, obese donors pose an additional operative risk to this procedure.

### Living donors with dyslipedemia

Various types of dyslipidemia are associated with decreased kidney function in the general population and have faster rates of progression in patients who have chronic kidney disease. However, isolated dyslipedemia is not a contraindication for donation.[9]

### Living donors with history of malignancy

The risk of clinical and subclinical malignancy increases with age, especially over 50 years. Currently malignancy is a contraindication for organ donation, except for low-grade non-melanoma skin cancer.[9]

A prior history of malignancy may be acceptable if prior treatment of the malignancy:

- Does not decrease renal reserve or place the donor at increased risk for ESRD.
- Does not increase the operative risk of nephrectomy.
- Has cured the cancer and is not potentially transmissible. Some of these would be colon cancer (Dukes A, >5 years ago), non-melanoma skin cancer or carcinoma *in situ* of the cervix.

Informed consent, however, should be obtained including discussion with both donor and recipient that transmission of malignant disease cannot be completely excluded.

## LIVING DONORS WITH POTENTIAL TRANSMISSIBLE INFECTIONS

HIV positive status is a contraindication for donation. Cytomegalovirus (CMV) and Ebstein-barr virus (EBV) status is measured at some transplant centers and they delay transplant till PCR for CMV becomes negative. Most of the adults are EBV and CMV-positive; most of the children are negative. The risk of post-transplantation lymphoproliferative disorder (PTLD) is the concern in CMV and EBV-negative individuals receiving positive donors. However, the risk is not as high to prohibit renal transplantation.[9] Hepatitis C Virus (HCV) positive donor may be considered for donation to a HCV positive recipient only if the donor PCR is negative, certain genotypes (Genotype 4) are treated and eradicated of the donor and there is no evidence of chronic hepatitis or cirrhosis on liver biopsy. The Spanish group has transplanted kidneys from cadaveric donors with HCV reactivity to HCV positive recipients.[39] However, there is no data on live kidney transplantation from HCV positive donors. Hepatitis B Virus (HBV) positive status currently is not accepted for donation. However, there are some isolated reports of transplantation by groups in New Zealand.[9] Past history of pulmonary tuberculosis is not a contraindication for donation. Potential donors with positive serology for syphilis should be confirmed with fluorescent treponemal antibody (FTA) absorption test. Donors with a positive confirmatory FTA should be treated according to stage and donation should be delayed until successful treatment is accomplished. Treated Schistosomiasis is not a contraindication for donation.

## ESTIMATION OF RISK FOR POTENTIAL LIVE DONORS WITH MEDICAL ABNORMALITIES

The risk assessment applies not only to donors with isolated medical abnormalities (IMAs) such as hematuria, low-grade proteinuria, hypertension, stone disease and borderline normal GFR[25] but also to “normal-criteria” donors. R. Steiner proposed that the risk of ESRD for many IMAs can be estimated semiquantitatively by knowing the prevalence of the IMA in the general population and the incidence of the kind of ESRD with which that IMA might be associated. Steiner's formula:

Yearly risk for risk factor A = (Yearly incidence of ESRD A) / (Prevalence of risk factor A)

However, this formula estimates the baseline two-kidney risk for ESRD. Predicting the effect of nephrectomy is also a problem for normal-criteria donors, as some of them will develop diabetic nephropathy or other forms of ESRD after donation, later in life. Though their risks for ESRD are often lower, even the normal-criteria donors need to know their risks.

## ETHICAL ISSUE IN ACCEPTING COMPLEX LIVING KIDNEY DONOR

When considering complex living donors one should remember basic ethical principles of beneficence to the recipient, non-maleficence regarding the donor and the donor's right to autonomy.[40] Beneficence implies an active attempt to advocate strongly for the best medical treatment for patients with ESRD (i.e., renal transplantation). The principle of non-maleficence means the notion that medical professionals have a duty to “do no harm”—which is threatened by a scenario in which living donors must undergo a surgical procedure with a range of substantial risks, including death.[41] In addition, living donors subject themselves to harm voluntarily, typically desiring to answer the need of another person, rather than gaining direct physical or financial benefit from their action. The judgment of the potential donor is not simple; donors often have complex motivations, beliefs and perceived obligations that may not be apparent and that may influence risk tolerance. For instance, a mother may be willing to tolerate a high level of personal risk because of her powerful desire to help her child. Some prospective donors are motivated by duty, love or religious conviction and are indifferent to risk. Should the risk and benefit be forced on them when accepting their decision of donation? The basic ethical problem in accepting complex living donors is the availability of only limited medical information about the magnitude of potential risk. If data about donor risk are misrepresented, then donor autonomy is undermined.

## LEGAL CONTEXT FOR ACCEPTING COMPLEX LIVING DONORS

Generally, courts have required organ donor consent to be informed by the disclosure of pertinent risks and benefits of the procedure. However, some courts have considered requiring a more rigorous standard of voluntariness in the informed consent process and have extended the disclosure requirements to include risks for future harm. We don't have enough sound medical data of future risks to the complex living donor and this could lead to legal problems.

## FUTURE STRATEGIES FOR COMPLEX LIVING KIDNEY DONATION

Resse PP[40] proposed potential strategies to approach complex living kidney donors. To maximize independence of donor evaluation, he proposed integration of ‘donor advocate’ to the transplant centers. The latter would be a non-partisan professional representing the donor's interest independently. He further suggested that potential donors should be made to contact previous donors with similar risk factors to improve informed consent process. Computer teaching software and simulators could enhance donor education and decision-making. He further recommended long-term outcome studies for complex donors with a planned follow-up after donor nephrectomy. This would not only help the complex donor but also the transplant society and public in understanding the future potential risks. Donors should be involved in the development of programs and research that are aimed at improving care.

We should remember that easing the donor criteria would not boost the availability of organs significantly. According to Martin Karpinski *et al*.,[42] accepting living donors with mild hypertension or proteinuria for kidney transplants would lead to only a modest increase in transplantation rates (3%). But every single additional transplant counts. The decision to accept a medically complex living donor rests on an uncertain medical, ethical and legal foundation, so transplant centers ultimately must tailor their decisions to the particular circumstances of each donor. Given the evidence of increasing media and public interest in the medical and ethical basis of living donation, transplant professionals would be well advised to make internal debates about complex donors more transparent. In the past several years, stories expressing concern about living donors have appeared in diverse media outlets. If public trust in transplant professionals were jeopardized, with an attendant decline in living donation, then the harm to potential recipients would be devastating.

## NON-HEART-BEATING DONOR (NHBD)

Kidneys transplanted from non-heart-beating donors (NHBD) are generally regarded as marginal or extended criteria grafts due to the associated period of warm ischemia. The concept of using organs from NHBDs for transplantation is not new. It was practiced in the 1950s and 1960s when clinical organ transplantation became a reality.[43] The outcomes were suboptimal. The legal definition of brain death was introduced and thus cadaveric organ donation from brain-dead patients became the most common and preferred source of organs for transplantation. However, with an increased demand for organs, the utilization of NHBDs as donor source was re-explored.

The NHBD is a donor who has suffered an irreversible brain injury (usually from trauma or stroke) but does not fulfill the criteria for brain death. The patient is pronounced dead only after sustained cardiac asystole, which results in prolonged warm ischemia and damage to the procured organs. This is in contrast to a standard cadaver organ donor who has been pronounced brain dead and the heart is still beating until the very moment of flushing of the donor organs with cold preservation solution. This method usually results in less stress in the procured organs. The First International Workshop on Non-Heart-Beating Donors was held in Maastricht, the Netherlands, in 1995.[44] The following consensus statements were issued: 1. NHBD organs need to be considered for transplantation. The use of NHBD organs can be a valuable way to increase the number of organs available for transplantation. 2. NHBD program should be started only after a written protocol is approved by the local medical ethics committee. 3. Similar to cadaver donors, the diagnosis of death in an NHBD has to be made by a physician independent of the transplant team. 4. Openness and public education regarding NHBDs are essential to prevent public mistrust.[44]

Teraoka *et al*.[45] reported on early graft function in 742 kidney transplants from NHBDs from the United Organ Sharing Network System in Japan. The average post-transplant dialysis was 13 days and 5% of kidneys never regained function. When compared to those donors who underwent cannulation before cardiac arrest for *in situ* perfusion, the warm ischemia time was short and there was decreased rate of nonfunction. However, no difference in serum creatinine was observed at one-year follow-up.

Yong Cho *et al*.[46] presented a multicenter review on 377 NHBD kidney transplants performed at 77 transplant centers in the US from 1995-1998 and compared the outcomes with 12,156 kidney transplants from brain-dead heart-beating donors (HBDs) during the same time period. Eighty-five of the NHBD kidneys were procured from uncontrolled donors and 279 were from controlled donors, there was no significant difference in one-year graft survival between the groups. Similarly, there was no difference in one-year graft function, regardless of whether the NHBD kidneys were procured with core cooling. Comparing the NHBDs with the HBDs, there was a significant increase in early dialysis (47% vs. 25%), primary nonfunction (3% vs. 1%) and serum creatinine at discharge (4.4 mg/dL vs. 3.2 mg/dL). Despite the inferior initial graft function of the NHBD kidneys, there were no significant differences in one- and three-year graft survival rates (85% and 73% in the NHBD group vs. 87% and 77% in the HBD group). Despite poorer early graft function, this data provided convincing evidence that NHBD kidneys yielded similar graft survival results to cadaveric kidneys and should be pursued to expand the donor pool.

Alanso *et al*.[47] presented a single-center study of kidneys obtained from NHBD, in a 14-year period from Spain (1989 and 2004). One hundred NHBD were compared to 1025 HBD transplanted during the same period. Delayed graft function and primary nonfunction was higher in the NHBD group (DGF; 84% vs. 26%; *P* <.001) and (PNF;16% vs 10%; *P* <.001) respectively. The short-term (three-month and one year) and long-term (five and 10 years) renal function, determined by the serum creatinine levels and patient and graft survival were not different for kidneys obtained from NHBD.

The NHB donors can make a significant contribution to transplant numbers. In Maastricht (Netherlands), 40% of transplants and in Leicester (UK) 22% of transplants are performed from NHB donors. However, in both centers there was no increase in the overall transplant rate and it was probably due to the fact that the effort and resources dedicated to these programs has resulted in a decrease of kidneys from other and potential sources.[29] In spite of all the benefits, transplants with NHBDs are in a plateau phase. UNOS data suggests that they formed 100 transplants a year over the last two decades, i.e. approximately 2% of cadaveric organs. Many transplant centers are reluctant to use kidneys from NHBDs due to relatively higher incidence of primary nonfunction (PNF). Issues like uncertainty regarding diagnosis of death on the basis of cessation of cardiac activity (cardiac death), logistics of family consent involved in the procurement of organs and prolonged warm ischemia all contribute to its slow development. It is important to educate the public, hospitals and physicians about the possibilities of organ donation from NHBDs. Public trust is most important in the success of any transplant program.

## MARGINAL CADAVERIC KIDNEY DONOR

Marginal cadaveric kidney donors can be defined as: all donors older than 60 years, donors older than 50 years with any of the following criteria: (1) hypertension, (2) cerebro-vascular cause of brain death or (3) pre-retrieval serum creatinine (SCr) level > 1.5 mg/dl, with a degree of glomerulosclerosis >15% and prolonged cold ischemia.

Transplantation with marginal cadaveric kidney donor is established. There is enough published evidence of its better outcome than waitlisted patients. The focus is on finding various ways to improve the outcome of such marginal grafts. In a consensus statement, an international panel of pathologists presented a methodology to as-sess the marginal kidneys based on the viable neph-rons to guide about single or dual transplantation or discard the organ.[48] This panel suggested a biopsy-based scoring system for kidneys, with scores ranging from a minimum of 0 (indicating the absence of renal lesions) to a maximum of 12 (indicating the presence of marked changes in the renal paren-chyma).[48–52] Kidneys with a score of 3 or lower were predicted to contain enough viable nephrons for single transplants. Those with a score of 4, 5 or 6 could be used as dual trans-plants, on the assumption that the sum of the viable nephrons in the two kidneys approached the number in one ideal kidney. Kidneys with a score of 7 or greater were discarded, since it was assumed that they would not deliver sufficient number of nephrons, for even dual transplanta-tion.[48–51] This hypothesis was tested in a recently published prospective cohort study.[53] Outcomes among 62 patients who re-ceived one or two histologically evaluated kidneys from donors older than 60 years of age were compared with the 248 matched recipi-ents of single kidney grafts that were histologically evaluated and were either from donors 60 years of age or younger (124 positive-reference recipients who, according to available data, were expected to have an optimal outcome) or from those older than 60 years (124 negative-reference recipients, expected to have a worse outcome). The primary end point was graft survival. The performance of pre-implantation histological evaluation predicted better survival both in the whole study group (*P* = 0.02) and among recipients of kidneys from older donors (*P* = 0.01). They concluded that the long-term survival of single or dual kidney grafts from donors older than 60 years of age is excellent, provided the grafts are evaluated histologically and allocated before implantation.

Younger renal transplant recipients often outlive their allografts, whereas older recipients often die before their allograft fails. This further adds to the waiting list for transplant. To improvise better utilization of marginal cadaveric kidneys a Deceased Donor Score (DDS) was used by Nyberg *et al*.[54] to assess graft survival. Rather than defining a donor as meeting expanded criteria or not, the DDS is a quantitative approach that provides a donor score on a continuum of 0-39 points and stratifies deceased kidneys into four grades (A, B, C, D). They reported a significant number of cadaveric donor kidneys (10.7%) which were identified by the DDS system as likely to have decreased post-transplant function and graft survival that were missed by the ECD system, demonstrating greater predictive ability of the DDS.

## CONCLUSION

With the ever-increasing pressure to accommodate more deserving renal transplant recipients and the related paucity of an active cadaver program, there is a tremendous pressure on the transplant surgeon to accept less than ideal living donors for organ donation. In our decision process, we need to consistently review ourselves of the Hippocratic oath “primum non nocere” to both recipient and donor.
